# Latine mothers' conceptions of *bien educado* in U.S. Latine adolescents: Respect, humility, good manners, and consideration for others as Core aspects

**DOI:** 10.1111/jora.70227

**Published:** 2026-07-06

**Authors:** Alysia M. Cruz, Gustavo Carlo

**Affiliations:** ^1^ Department of Educational Psychology Texas A&M University College Station Texas USA; ^2^ School of Education University of California, Irvine Irvine California USA

**Keywords:** bien educado, cultural values, Latine families, parenting practices, prosocial development

## Abstract

Developmental theories of ethnocultural socialization (Incorporating the cultural value of respeto into a framework of Latino parenting. *Cultural Diversity and Ethnic Minority Psychology*, 16(1), 77–86; Traditional and culture‐specific parenting of prosociality in U.S. Latino/as. *Oxford handbook of parenting and moral development*, Oxford University Press.) emphasize the importance of understanding how caregivers instill culturally grounded values adapted to specific cultural and environmental contexts to support positive youth development. Caregivers are deemed to transmit these cultural values to their youth via ethnic heritage practices, customs, rituals, and cultural experiences. Importantly, work based on these models has shown that specific cultural values significantly predict the health and well‐being of Latine youth (Cultural values, empathy, and prosocial behavior among U.S. Latino/a youth: A mediation model. *Journal of Social and Personal Relationships*, 38(2), 521–540; Familism values and adjustment among Hispanic/Latino individuals: A systematic review and meta‐analysis*. Psychological Bulletin*, 147(9), 947–985). Among the cultural values instilled by many Latine parents is *bien educado*, a concept that emphasizes the importance that youth be well‐mannered, display positive social behaviors, and exhibit a strong moral character (Familismo, respeto, and bien educado: Traditional/cultural models and values in Latinos. *Critical Cultural Studies of Childhood*. Springer). While much of the prior research has focused on cultural values such as *familismo* and *respeto*, relatively little is known about the cultural value of *bien educado*. Indeed, no studies exist on how *bien educado* is conceptualized and taught by Latine mothers. To address this gap, focus group interviews were conducted to explore how Latine mothers of adolescents define and teach *bien educado* to their adolescents. Seventeen Latine mothers (*M*_age_ = 41.88 years, *SD* = 6.23) reporting on their adolescents (*M*_age_ = 14.97 years, *SD* = 3.55) participated. On average, mothers had two children and lived in the United States for approximately 21.6 years. The majority were of Mexican origin and predominantly Spanish‐speaking (88%). Focus group interviews were conducted in Spanish and English using semi‐structured questions designed to explore mothers' definitions, beliefs, and practices related to *bien educado*. The questions included: “What is your own definition of *bien educado*?” and “What are some things you do to teach your child to be *bien educado*?” All focus groups were audio‐recorded, transcribed verbatim, and trained; bilingual coders analyzed the transcripts using inductive thematic analysis (*Qualitative inquiry and research design: Choosing among five approaches*. SAGE Publications). Mothers conceptualized *bien educado* as encompassing four main aspects: respect, consideration for others, humility, and being well‐mannered. Furthermore, mothers emphasized their intentionality to foster these traits in their youth and viewed the concept as a strength‐based approach that contributed positively to their youth's moral development. The findings reveal that *bien educado* is a multidimensional cultural value construct and a Latine culturally‐relevant parenting goal. Specifically, the findings highlight the central elements of *bien educado* as respect, consideration for others, being well‐mannered, and humility. Given the centrality of these four elements in prosocial and moral development (The development and correlates of prosocial moral behaviors. *Handbook of moral development*. Psychology Press.), the present findings situate *bien educado* as a Latine‐grounded value that can further our understanding of U.S. Latine youth prosocial and moral development. Moreover, the findings demonstrate the need to incorporate this understudied core Latine cultural value into sociocultural‐ and ecological‐grounded parental socialization models and future research in U.S. Latine youth.

Across diverse cultures, parents aspire for their children to develop and exhibit prosocial traits such as empathy, respect, kindness, and strong moral qualities that are essential for healthy, well‐functioning societies, communities, and individuals. Previous research has shown that youth who exhibit high levels of prosocial behavior tend to have better psychological health, improved academic outcomes, and reduced aggression and antisocial behavior (Carlo, 2014).

Recognizing the significance of prosocial development for both individual and community well‐being, scholars have increasingly used cultural frameworks to understand how these traits are cultivated within specific sociocultural contexts (Calzada et al., 2010; Knight & Carlo, 2012). These frameworks emphasize how parents transmit culturally grounded values and behavioral expectations that shape children's social competence and moral development. Specifically, cultural scholars have focused on the values, beliefs, and daily practices that Latine caregivers and families teach to influence youth moral and prosocial development (Carlo & Conejo, 2019). Moreover, these researchers identify the multifaceted ways that cultural traditions, customs, and cultural mechanisms inform youth identity formation, foster cultural competence, and promote positive mental health and overall well‐being (Carlo, 2014). For example, scholars have identified several specific core cultural values endorsed by most Latine parents, such as respect, familism, and obedience (Cahill et al., 2021; Calzada et al., 2010). These interconnected values are believed to have a significant influence on the development of ethnic identity and adjustment in Latine youth (Streit et al., 2020). Accordingly, research suggests that parents attempt to instill values early, allowing children to integrate them alongside broader cultural and social influences.

Although cultural constructs such as respect and familism have received significant scholarly attention in Latine research, less is known about other culturally embedded mechanisms that may similarly influence prosocial and moral development in Latine youth. One such mechanism is the cultural value of *bien educado*, a multidimensional concept that emphasizes how Latine families conceptualize and encourage positive social behavior, moral integrity, and respect for others. Prior literature on *la educación* [the education] emphasizes moral education, referring to the development of a child's ability to distinguish right from wrong and to make morally appropriate decisions (Prins, 2011). This dimension highlights that beyond behavioral compliance, cultural socialization also involves fostering internalized moral reasoning and ethical judgment (Carlo, 2014). However, *bien educado* remains understudied, despite its close ties to key elements of positive youth development (Bridges et al., 2012). Exploring *bien educado* provides an opportunity to extend current understandings of Latine cultural values and to highlight how parents actively transmit these ideals across generations.

As noted, *la educación* [the education] refers to a broader cultural framework that encompasses not only formal schooling but also moral upbringing, social behavior, and the development of culturally appropriate values. The process of *educar* [to educate] refers to how parents and caregivers socialize children through daily interactions, modeling, and guidance. Within this framework, *bien educado/a* [well educated] is used as an adjective to describe individuals who embody these culturally valued prosocial behaviors and moral qualities, whereas *mal educado/a* [poorly educated] refers to individuals who demonstrate the antithesis of prosocial and positive moral qualities. Although *bien educado* is grammatically gendered in Spanish, we use the term throughout the manuscript as a general construct label for consistency.

## *Bien educado* as a cultural mechanism

*Bien educado* is deemed to encompass several Latine prosocial and moral traits, including respect, consideration of others, well‐mannered behaviors, and helpfulness. These are traits that include responsivity to the need of others and generosity, which go beyond obedience to authority and decorum (Calzada et al., 2010; Carlo & Conejo, 2019). However, some scholars, such as Bridges et al. (2012), conceptualize *bien educado* as a Latine construct that encompasses a broader range of attributes that include composure, respect, obedience, cooperation, and meeting role obligations (Bridges et al., 2012).

Although *bien educado* is conceptually related to several values and traits, such as respect, it reflects a broader aspect of cultural functioning. *Respeto* [respect] is typically understood as an internalized value orientation that guides how individuals believe they should treat others, particularly authority figures (Calzada et al., 2010; Halgunseth et al., 2006). In contrast, *bien educado* captures how those values are expressed and, importantly, recognized by others. In this way, *bien educado* reflects a socially perceived outcome of early cultural socialization, one that signals that an individual has been raised to engage in culturally appropriate, respectful, and morally grounded ways across contexts. Rather than referring to discrete behaviors or isolated traits, *bien educado* reflects a broader evaluation of the individual's character and upbringing, shaped through consistent patterns of interaction over time.

Although it also overlaps with Latine relational values such as familism, which emphasize family obligation, support, and connectedness (Knight et al., 2010) and observable prosocial behaviors (e.g. helping and sharing; Carlo, 2014), it is not reducible to these constructs. Instead, it operates at the intersection of cultural values, behavioral expression, and social perception, reflecting not only how individuals conduct themselves, but how they are perceived as embodying culturally valued norms of conduct within Latine culture.

The acquisition of these positive attributes involves parents and family members socializing their children to foster individuals who embody the value of *bien educado*. Given its importance, there is a critical need to explore the role of *bien educado* in the development of Latine youth and to understand the specific conceptions of this value within this cultural context. While extensive research on Latine populations has focused on other cultural aspects, such as familism, ethnic identity, and respect, *bien educado* remains a construct that has been significantly understudied.

Although *bien educado* is often introduced in early childhood through expectations of manners of respect, manners, and appropriate social conduct (Bridges et al., 2012), its meaning, expressions, and expectations may become more nuanced during adolescence as youth navigate peer relationships, social pressures, and evolving cultural identities. Importantly, the meaning and expression of *bien educado* may shift across developmental periods, particularly during adolescence. This stage is marked by increased autonomy, identity exploration, and greater engagement with social contexts beyond the family, all of which shape how youth interpret and enact culturally grounded values (Fuligni & Tsai, 2015). During this time, youth are actively navigating cultural and societal expectations while forming a sense of self, making parental socialization particularly important (Smetana et al., 2015). For example, demonstrating respect and consideration for others may extend beyond obedience to authority (Calzada et al., 2012) and instead involve more complex forms of perspective‐taking, moral reasoning, and socially appropriate behavior across diverse contexts.

As such, understanding how parents conceptualize *bien educado* is particularly important for capturing how this cultural value may guide adolescents' social behavior and adjustment. This focus is especially critical during adolescence, as youth are increasingly required to navigate cultural expectations across home, peer, and institutional contexts. Thus, making culturally grounded values central to their social decision‐making and identity development (Roche et al., 2014). More broadly, examining *bien educado* through caregivers' perspectives aligns with calls for culturally grounded approaches to parenting by centering parent‐defined values and socialization goals within Latine families.

## Parents as primary socializing agents

Parents and caregivers play a central role in transmitting cultural values and knowledge, making them the primary socializing agents in developing *bien educado*. Often, this value is taught and demonstrated at home, where parents model good behavior, instruct their children to be polite to others, show respect for elders and authority figures, and encourage courteous interactions. By instilling these behaviors and traits in their daily interactions, parents communicate moral expectations to their children. They not only tell their children to be *bien educado* but also demonstrate this value through their actions and treatment of others. In doing so, they model what it means to be a good person (*una persona bien educada*), demonstrating self‐control, empathy, respect, and consideration for others.

Importantly, this process is well‐aligned with the broader literature on familial ethnic socialization, which emphasizes the role of parents transmitting culturally relevant values through ethnic‐racial socialization processes, beliefs, and behavioral expectations to their children (Hughes et al., 2006). Within this framework, cultural socialization practices, including the promotion of cultural traditions, norms, and interpersonal values, serve as central mechanisms through which children learn how to function as competent members of their cultural group. Among Latine families, these practices often involve the explicit and implicit teaching of values such as respect, familism, and proper social conduct (Calzada et al., 2012).

Building on this perspective, cultural values transmission theory offers a theoretical framework that focuses on how cultural norms, values, practices, and beliefs are transmitted intergenerationally through social learning and modeling (Carlo & Conejo, 2019). Culture, knowledge, and beliefs are also sustained and transmitted through parental influence and, in turn, are thought to shape parenting practices that help children acquire knowledge of their culture and become culturally competent members of their society (Bornstein, 2012). Similar to cultural transmission, social learning and modeling are central mechanisms of the parenting socialization model (Calzada et al., 2012). Children learn by observing and imitating the behavior of their parents, which facilitates the transmission of cultural knowledge and practices. The parent socialization model also emphasizes the role of parenting styles and practices in imparting this knowledge. These practices then influence children's social development and emotional well‐being.

As children internalize these cultural values, they become a fundamental part of their identity (Quintana & Scull, 2009). Demonstrating these values aligns with the family's cultural expectations. Because of this, in Latine culture, it is very important for children to exhibit good behavior for the family's overall functioning and *representación de la familia* (representation of the family). Therefore, the family's reputation extends beyond just the home, and when children consistently demonstrate the principles of *bien educado* in different social settings, this not only reflects well on their personal character but also enhances the family's image within the community (Lopez et al., 2022). As a result, families are viewed as embodying positive social values and cultural awareness within the broader community.

However, when children do not show the qualities of *bien educado* and behave disrespectfully, selfishly, and transgressively, they are judged by outsiders as being *mal educado* or *sin educac*ió*n* [poorly educated or not educated] (Purcell‐Gates & Bustos Rojas, 2009). Failing to demonstrate these values also is often viewed as a sign of poor parenting skills (Leon, 2010). Sometimes, *bien educado* is contrasted with the concept of *mal educado*. Thus, the distinction between a *bien educado* and *mal educado* youth is a subjective judgment that can differ depending on cultural and personal views of what is considered socially considerate and polite.

While parents play a central role in socializing their children, shaping their cultural values (Grusec, 2002; Grusec & Davidov, 2019), and transmitting the concept of *bien educado*, it is essential to recognize that the family's influence is not unidirectional. Instead, both parents and children contribute to and are influenced by the shared cultural values and practices within the family context. Consistency in reinforcing values, expectations, and positive behaviors is also a key factor in instilling *bien educado* within the family. Thus, when parents consistently model and reinforce these positive attributes (e.g., positive self‐conduct, strong moral character) (e.g., Carlo & Conejo, 2019), values (e.g., familism, obedience, respect), and expectations (e.g., remaining humble), this creates a home environment where being *bien educado* becomes an essential part of daily living (Calzada et al., 2010, 2012).

Despite these conceptual scholarly discussions, little is known about how Latine parents define *bien educado*. Although prior work highlights the role of parents as primary socializing agents, research has not directly examined how mothers conceptualize this construct or whether these definitions vary across cultural contexts. One potential dimension through which such variation may emerge is language use. Among Latine families, language preference (e.g., Spanish vs. English) is often closely tied to cultural orientation, acculturation, and the retention of core cultural values (Calzada et al., 2012; Knight et al., 2010). Prior research suggests that Spanish‐speaking parents may be more likely to endorse and emphasize culturally rooted values such as familism, respect, and interdependence, whereas English‐speaking parents may reflect greater exposure to and integration of mainstream U.S. cultural norms (Cruz et al., 2025). These patterns may shape how parents conceptualize and transmit culturally embedded constructs such as *bien educado*. Accordingly, the present study considers whether mothers' definitions of *bien educado* may vary as a function of language use, with these observations treated as exploratory.

## THE PRESENT STUDY

There is one prior empirical study on the concept of *bien educado* (Bridges et al., 2012). In this prior work, a measure of *bien educado* was developed and validated using parent and teacher reports of young Mexican‐heritage children's social behaviors within early educational settings. This work represents an important contribution by offering an initial operationalization of the construct in childhood. However, because this approach relied on adult reports of children's observable behaviors, it primarily captures how *bien educado* is enacted rather than how it is conceptualized by caregivers themselves. As such, less is known about the meanings parents attribute to *bien educado* and the cultural logics that guide its socialization.

To address this gap, the present study used qualitative methods (i.e., focus groups) to capture how culturally embedded values are understood by Latine mothers. Using a culturally grounded approach, we investigated how mothers define the construct of *bien educado* in their own words. Given prior work suggesting that cultural orientation and value transmission may vary across Latine subgroups and linguistic contexts, we also explored whether conceptualizations of *bien educado* varied by language use. Specifically, the study included Latine mothers, the majority of whom were of Mexican origin, as well as Spanish‐ and English‐dominant mothers. We hypothesized that Latine mothers would describe *bien educado* using themes such as being well‐mannered, treating others with respect, being considerate, and possessing humility. Given the small number of English‐dominant mothers, language‐related observations were treated as exploratory.

### Positionality Statement

As researchers, it is important to critically reflect on our positionality and how our identities and experiences may shape the research process. The first author identifies as a U.S.‐born, bilingual, Dominican and Puerto Rican woman. While she shares cultural ties with the broader Latine community represented in this study, she acknowledges that she is not a member of the specific families or local communities from which the data were collected. Her interest in the cultural value of *bien educado* is rooted in both personal lived experience and scholarly commitment, shaped by her upbringing in a bicultural and bilingual household and a deep respect for how Latine families transmit cultural values intergenerationally. The first author recognizes that her own experiences and beliefs, particularly the value she places on family, respect, and cultural socialization, may influence her approach to the research.

The second author identifies as a native‐born, Puerto Rican, bilingual man who endorses many common core Latine cultural values and beliefs, but who is also not a person from the Mexican heritage culture represented in the present study sample. However, the concept of *bien educado* is a lived experience and deeply rooted in his belief system. He has also extensively studied ethnic and racial identity, cultural values, and prosocial and moral development in mostly Mexican heritage samples. Additionally, the research team consisted of graduate student colleagues of Mexican heritage and an undergraduate researcher of Mexican heritage.

To ensure reflexivity and reduce potential bias, the team engaged in ongoing dialogue with a small research team throughout the data analysis process. The team critically discussed and resolved differences in emerging themes, challenged assumptions, and worked collaboratively to ensure that the findings remained grounded in the study participants' perspectives and Latine cultural contexts.

## METHOD

The present study used a qualitative approach to explore how Latine mothers define the cultural Latine value of *bien educado*. To better understand the complexities of how this core cultural construct, focus group interviews were conducted, allowing participants to describe their understanding of *bien educado*.

### Research design and participants

Participants were 17 Latine mothers (*M*_age_ = 41.88, *SD* = 6.23) reporting on their adolescents (*M*_age_ = 14.97, *SD* = 3.55). Mothers reported the ages of all children in the household; however, the reported mean age reflects adolescents within the target age range. Participants were recruited from a community center in Orange County, California and were recruited specifically for this focus group study and were not part of a larger dataset. The majority of participants were Spanish‐speaking (*n* = 14), with a smaller number of English‐dominant mothers (*n* = 3), limiting meaningful comparisons between groups. Most participants were of Mexican origin and predominantly Spanish‐speaking (88%), with a small number of mothers representing other Latine backgrounds (e.g., Colombia, El Salvador). The majority of mothers were foreign‐born and lived in the United States for approximately 21.6 years. On average, mothers had two children. Educational attainment varied across participants, ranging from primary education and university degrees.

Mothers were then asked a series of questions to examine and assess the value of *bien educado*. Focus group interviews were audio recorded, transcribed, and qualitatively coded for themes related to *bien educado*. The coding of these interviews also utilized reliability coding, in which 30% of transcripts were randomly selected and coded by a trained second coder. Any discrepancies were resolved by discussion.

### Focus group interview procedures

Data for this study were collected in January 2025 as part of a standalone qualitative investigation examining Latine mothers' conceptualizations of *bien* educado. Participants were recruited through community‐based partnerships established via the Outreach and Community Engagement Network (OCEAN) at the University of California, Irvine. OCEAN collaborators referred the research team to a local community center that serves Latine families, where recruitment took place. Recruitment methods included flyers and sharing study information through community networks and center staff.

To be eligible, participants had to (a) self‐identify as Latine, (b) be a mother or primary caregiver of at least one child, and (c) be able to participate in either English or Spanish. The community center was selected as the data collection site due to its accessibility and its role as a trusted, culturally relevant space for Latine families. This study received approval from the University of California, Irvine. Institutional Review Board (IRB), and all participants provided informed consent prior to participation.

Data were collected through focus group interviews, defined as small‐group discussions in which multiple participants engaged in shared conversation guided by a trained moderator. A total of three focus groups were conducted. Each session lasted approximately 60 min and was held in a private room at the community center.

The focus groups were facilitated by a trained bilingual researcher who identified as Latina, with prior experience in qualitative data collection and conducting focus groups with Latine families. The facilitator had graduate‐level training in developmental and cultural research and was familiar with the construct of *bien educado* as well as culturally responsive interviewing practices. This shared cultural and linguistic background helped foster rapport and encourage open discussion among participants. The moderator followed a semi‐structured interview guide designed to elicit mothers' perspectives on *bien educado*, while allowing flexibility for participants to share their experiences. All sessions were audio‐recorded with participants' consent.

The interview protocol included open‐ended questions assessing familiarity with *bien educado*, its meaning, importance, and how it is socialized within families. Mothers were also asked to describe behaviors reflecting *bien educado* and *mal educado*. (1) What is your own definition of *bien educado*? (2) How important is *bien educado*? (3) How do you know that your child is *bien educado*? (4) What are some things that you do to teach your child to be *bien educado*? (5) What about the concept of being *mal educado*? (6) What does it mean to be *mal educado*?

All focus group interviews were audio‐recorded and transcribed verbatim. Spanish focus group interviews were transcribed by a native Spanish speaker and thoroughly verified for accuracy by a second native Spanish speaker. Transcripts were then translated into English, and all resulting transcripts (both English and Spanish) were double‐checked for accuracy by a second transcriber. Pseudonyms were used to protect participant confidentiality.

#### Data analytic approach

Focus group data were analyzed using an inductive thematic analysis approach to identify mothers' conceptualizations of *bien educado*. Responses were reviewed line by line to generate initial codes, which were then refined and organized into four overarching themes capturing the core ways mothers described this construct. Given the study's interest in potential variation by language use, mothers were initially grouped by language dominance (Spanish‐dominant and English‐dominant). However, because the majority of participants were Spanish‐dominant, meaningful comparisons were not feasible. Accordingly, analyses focused on identifying and interpreting the central themes across the full sample to capture shared understandings of *bien educado*, with language‐related observations treated as exploratory.

### Interview data coding

To answer the research question regarding mothers' definitions of *bien educado*, focus group interviews were coded using an iterative, inductive approach. Data were transcribed verbatim and analyzed through close reading, memoing, and repeated engagement to identify patterns, nuances, and culturally grounded meanings. This process supported a culturally sensitive interpretation of how Latine mothers define and transmit the value of *bien educado*.

An inductive coding approach (Creswell, 2013) was used to identify patterns, themes, and categories within the data without using predetermined theoretical frameworks. During the first phase of coding, an iterative process was employed to categorize themes. The coding process was conducted by a team consisting of the study authors and trained research assistants with experience in qualitative methods. The team collaboratively developed and refined the coding scheme through regular meetings, during which discrepancies were discussed and resolved by consensus. Through this process, four overarching themes were identified from the focus group data.

To assess coding reliability, a trained secondary coder independently coded 30% of the transcripts using the inductively developed coding scheme. Interrater reliability, calculated using percent agreement, indicated that raters showed 88% agreement for well‐mannered, 88% for respect, 83% for humility, and 100% for consideration for others, reflecting overall consistency across coding categories. Any discrepancies were resolved through discussion, and the coding scheme was refined prior to coding the full dataset.

## RESULTS

### Latine mothers discuss dimensions of *bien Educado*

To explore how Latine mothers define *bien educado*, we analyzed focus group data to identify recurring themes in their descriptions and examples. The following sections detail these themes, supported by excerpts that illustrate how mothers define and conceptualize the cultural value of *bien educado*.

#### Dimensions of bien Educado

The major themes of *bien educado* that emerged from these focus group interviews included consideration for others, respect, humility, and being a well‐mannered person (see Table 1).

**TABLE 1:** Latine mothers' conceptions of bien educado.

Theme	Definition of theme	Sample excerpts from focus groups
Respect (*Respeto*)	A multifaceted value that encompasses obedience to authority, deference, appropriate public behavior, and interpersonal decorum. In Latine culture, *respeto* plays a central role in maintaining harmony within families and communities by reinforcing social hierarchies and promoting collective well‐being. It involves showing high regard for elders and authority figures such as parents, grandparents, and teachers and reflects a broader cultural commitment to upholding order, stability, and mutual support across generations.	*Lo que venga en casa. El ejemplo. Yo pienso que sí, el ejemplo está bien, pero también uno ya trae también esa educación de sus papás. A veces también ya tienes eso, que tus papás te enseñaban, te decían, eso está bien, respeta a tus mayores, haz esto, haz lo otro, y uno trata también de enseñarle lo que uno trae a sus hijos. O sea, sé así, haz esto, respeta a las personas, respétate a ti mismo, y todo eso es lo que hacen*.
Consideration for Others (*Consideración hacia los demás*)	Refers to the practice of thoughtfully taking into account others' feelings, needs, and perspectives when making decisions and engaging in actions. It involves perspective‐taking, demonstrating genuine concern for others' well‐being, and fostering an environment where individuals feel valued, welcomed, and understood. This attribute includes being inclusive, offering support when needed, and showing kindness and thoughtfulness in one's interactions with others.	*You arrive at a place and you have to be respectful to people, greet each other, be a little more friendly, and, as they say, it's not just because a person has degrees or not, education is always the value that one shares with one's children*.
Humility (*Humildad*)	An individual who is *humilde* typically possesses an honest and modest attitude toward others, lacks arrogance or excessive pride, is unpretentious, refrains from attempting to impress others, and demonstrates respect for others. This attribute also describes someone who displays a sense of humility in their actions and the way they interact with others.	*Education opens doors for you, but more than anything, the humility to understand people. Because when you have an education, you can understand different cultures or whatever, because your education leads you to understand and do this. I mean, more than anything, to communicate with people, right? I mean, because you have an education, you know you can talk. Or communicate with different people. I think that's what education is: having empathy, humility*.
Well‐Mannered (*Buenos Modales*)	Refers to displaying respectful, polite behavior that aligns with socially accepted norms and etiquette. In Latine culture, being well‐mannered goes beyond individual conduct. It reflects a deep cultural emphasis on showing courtesy, deference, and consideration in interactions with others. This includes using formal language (e.g., *usted* when addressing elders), offering warm greetings, and practicing politeness in both speech and behavior.	*For me, as my colleagues have already said, education begins at home. Even one can educate oneself a little more, start teaching values to one's children, so they learn to respect one's ideas, the ideas of others, and I think respect is also a bit of that, respect. You arrive at a place and you have to be respectful to people, greet each other, be a little more friendly, and, as they say, it's not just because a person has degrees or not, education is always the values that one shares with one's children*.

*Consideration for others*. One dimension of *bien educado* Latine mothers consistently described was the importance of teaching their children to be considerate of others. Mothers emphasized raising children who are mindful of how their actions and words affect those around them. Being considerate also referred to treating others with empathy and warmth, and creating an environment where everyone feels welcome and valued. Many mothers emphasized the idea that being considerate is not only about etiquette or politeness, but also about fostering a deeper moral orientation, one that teaches children to be responsive and compassionate in various social contexts. Mothers often described this value in everyday examples. For example, one mother described how children are expected to actively help others and be attentive to those around them:

*I think maybe I'm not sure if it's in my family, but, yeah, like, estamos aquí y vas a ayudar. You're going to help whoever it is. Like, if you have a guest, like, you need this, you need that. What can I do for you? Like, how can we work together? It's like about the community of where you're at in that moment*.

These reflections suggest that *consideración* (consideration) refers to both social and emotional skills, deeply tied to Latine cultural values such as *personalismo* (personalism, warm interpersonal relationships) and *respeto* (respect), and that mothers see themselves as central to modeling and transmitting this value. One English‐speaking mother shared:

You arrive at a place and you have to be respectful to people, greet each other, be a little more friendly, and, as they say, it's not just because a person has degrees or not, education is always the value that one shares with one's children.

In this way, *consideración* (consideration) becomes an outward expression of an individual's upbringing and family values, evidence of what mothers described as true *educación*. It is not just about knowing how to conduct oneself, but about embodying respect and empathy in a way that reflects the values taught in the home. The belief that raising thoughtful children is a personal and collective duty that maintains traditions was strengthened by mothers' view of these actions as expressions of moral integrity and cultural pride.

*Respect*. The second dimension of *bien educado* described by Latine mothers was the value of *respeto* [respect]. Mothers consistently emphasized that *respeto* is a value that begins at home and is central to how they raise their children to be *bien educados*. Several participants described *respeto* as a foundational value that guides how children interact with others across contexts.

One mother explained:

*Para mí, como dijeron ya las compañeras, la educación empieza desde la casa, incluso uno mismo también puede educarse un poquito más, empezar a enseñarle valores a sus hijos, que aprendan a respetar sus ideas, las ideas de las demás personas y respetar también pienso que es un poco eso, respeto. Llegas a un lugar y tienes que ser respetuoso con las personas, saludarse, ser un poquito más simpático y, como dicen, no porque una persona tenga títulos o no lo tenga, la educación siempre son los valores que le disfruta a uno también a sus hijos*.

(English Translation)*: For me, as my colleagues have already mentioned—education begins at home. In fact, we can even take it upon ourselves to become a little more educated—to start instilling values in our children, teaching them to respect their own ideas as well as the ideas of others. I believe that is essentially what it comes down to: respect. When you enter a space, you must be respectful toward the people there—greeting them, being a little more personable. And, as the saying goes, regardless of whether a person holds academic titles or not, true education always consists of the values that one upholds and passes on to one's children*.

This perspective highlights how mothers link *respeto* to both internal values and outward behaviors, including greeting others, being polite, and treating people with dignity regardless of status. Other mothers emphasized that *respeto* is especially important in navigating differences in social interactions. As one participant shared:

*Like you know treat everyone with respect you know they might like things that you don't like but we have to treat start with respect*.

This example reflects how mothers described respect as a baseline expectation in social relationships, even when differences in opinions or preferences arise. Mothers also described respect as being demonstrated through specific behaviors such as being warm and polite and showing consideration in everyday interactions. Respectful behavior, such as greeting elders, listening without interrupting, and displaying humility, was described as a clear reflection of the values families work to instill in their children. Latine mothers in this study saw teaching *respeto* as a part of a larger duty to uphold their cultural values against other influences that could weaken or undermine them. Finally, one Spanish‐speaking mother shared:

*Lo que venga en casa. El ejemplo. Yo pienso que sí, el ejemplo está bien, pero también uno ya trae también esa educación de sus papás. A veces también ya tienes eso, que tus papás te enseñaban, te decían, eso está bien, respeta a tus mayores, haz esto, haz lo otro, y uno trata también de enseñarle lo que uno trae a sus hijos. O sea, sé así, haz esto, respeta a las personas, respétate a ti mismo, y todo eso es lo que hacen*.

(English Translation): *Whatever comes from home. The example. I think so, yes, the example is good, but you also have that upbringing from your parents. Sometimes you already have that, your parents taught you, they told you, that's good, respect your elders, do this, do that, and you also try to teach your children what you bring. I mean, be like this, do this, respect people, respect yourself, and all that's what they do*.

Across participants, *respeto* was consistently described as both a moral value and a set of everyday behaviors that reflect a child's upbringing.

*Humility*. In addition to *respeto* [respect], mothers highlighted humility as a central value they aimed to cultivate in their children. Humility was described as an orientation toward others rooted in empathy, openness, and a willingness to connect with others across differences. Mothers emphasized the importance of encouraging their children to keep an open mind, appreciate different viewpoints, and remain grounded no matter how successful they were. One mother reflected, suggesting that intellectual growth without humility lacks the relational depth needed to build meaningful connections. For her, *educación* was not just about acquiring knowledge, but about gaining the emotional and cultural awareness to communicate with others, especially those from different backgrounds, with empathy and respect. For example, one mother shared:

*Todo este tipo de cosas creo yo que habla de lo que es la educación ya más adelante como lo dice la compañera es el hecho de los títulos, el nivel educativo que tienes es diferente pero sí vale la pena hacer la separación porque a veces hay personas que sí tienen sus títulos y sus diplomas pero no tienen ni la educación ni la empatía para hablar con una persona que aunque no los tenga puede estar mejor educado*.

(English Translation): *I believe that all these kinds of things speak to the true nature of education. As my colleague mentioned earlier, the matter of degrees—the formal educational level one possesses—is a distinct issue; however, it is certainly worth making this distinction. For at times, there are individuals who indeed hold degrees and diplomas, yet lack both the education and the empathy to interact with someone who, despite not holding such credentials, may actually be better educated*.

This example highlights how mothers distinguished between formal education and the relational qualities associated with humility, particularly empathy and the ability to engage respectfully with others across differences. Mothers also described humility as closely tied to communication and understanding in diverse social contexts. As one participant shared:

*La educación te abre puertas, pero más que nada la humildad de entender a las personas porque cuando tienes educación puedes entender diversas culturas o lo que sea porque tu educación te lleva a entender y a hacer este, o sea, más que nada tener comunicación con la gente, ¿no? O sea, porque tienes educación y sabes que puedes platicar. O comunicarte con diferentes personas. Yo pienso que esa es la educación, tener empatía, humildad*.

(English Translation): *Education opens doors for you, but more than anything, the humility to understand people. Because when you have an education, you can understand different cultures or whatever, because your education leads you to understand and do this. I mean, more than anything, to communicate with people, right? I mean, because you have an education, you know you can talk. Or communicate with different people. I think that's what education is: having empathy, humility*.

Across participants, humility was consistently described as a key component of *bien educado*, reflected in the ability to listen, relate to others, and engage with others in modest and meaningful ways.

*Well‐mannered*. Mothers also emphasized the importance of raising well‐mannered children as a visible reflection of their home environment and moral education. Being well‐mannered included behaviors such as saying *please* and *thank you*, greeting others appropriately, using polite language, and showing consideration in social settings. As one participant explained:

*Seguir pautas de acuerdo al lugar donde estamos. Con fusil. Mis hijos, este, le digo, estamos en un parque, es tiempo de correr, jugar, gritar. Estamos en una biblioteca, es tiempo de guardar silencio, leer. Es acorde a las etiquetas del lugar donde estamos. Ajá*.

(English Translation): *We follow guidelines appropriate to our surroundings. I tell my children, We're in a park, this is the time to run, play, and shout. We're in a library, this is the time to be quiet and read. It's about following the etiquette of the place we are in*.

Several mothers shared that when their children behaved with courtesy and self‐control, it affirmed that they had been raised with proper values. For example, one Spanish‐speaking mother said:

*Por nosotros en la casa pueden ser de una manera y yo por ejemplo en la escuela me dicen felicidades señora, sus hijos son muy educados y yo digo, adiós porque en la casa bromean y lo que sea pero es bonito recibir eso de tus hijos que te digan, son bien educados y se portan bien a lo mejor no tienen una educación que digan, siempre te están saludando pero respetan, saben respetar a sus compañeros y se respetan a ellos mismos y es bonito eso que sepan respetar todo esto*.

(English Translation): At home, they might act one way. For instance, they joke around and do whatever, but when I go to school, people say to me, "Congratulations, ma'am; your children are very well‐mannered." It's quite a contrast! It is truly wonderful to hear that feedback about your children—to have people tell you that they are polite and behave themselves. Perhaps they don't have the kind of formal training where they are constantly greeting everyone they meet, but they do possess respect; they know how to respect their classmates, and they respect themselves. It is a beautiful thing that they understand how to show respect in all these ways.

Manners were not superficial behaviors but expressions of deeper values tied to cultural identity, family pride, and the social skills necessary for their children to thrive in diverse environments.

For the Latine mothers in this study, teaching their children to be well‐mannered was about much more than encouraging polite behavior. This instruction also consisted of instilling lifelong values that reflect care and self‐awareness. Manners were viewed as everyday practices through which deeper values such as respect, humility, and empathy are made visible. Being well‐mannered functioned as both a personal and social signal, communicating that their child has been raised with intention and discipline.

At the same time, mothers' descriptions also revealed an important distinction between being respectful and being well‐mannered. Although respect and being well‐mannered were often discussed together by mothers, they represent related but distinct elements of *bien educado*. Respect reflects a broader value orientation that guides how individuals view and relate to others, particularly in terms of acknowledging others' worth and maintaining appropriate social boundaries. In contrast, being well‐mannered reflects the behavioral expression of these values in everyday interactions, such as greeting others, using polite language, and demonstrating consideration of others. In this way, well‐mannered behavior can be understood as one mechanism through which respect is enacted. The overlap between these themes in mothers' descriptions highlights how values and behaviors are closely intertwined within the construct of *bien educado*, while also suggesting that respect provides the underlying motivation and well‐mannered behavior reflects its outward expression.

## DISCUSSION

This study explored Latine mothers' definitions and beliefs about the cultural value of *bien educado*. Across participants, *bien educado* was conceptualized as a multifaceted cultural value encompassing consideration for others [*consideración hacia los demás*], respect [*respeto*], humility [*humildad*], and being well‐mannered [*buenos modales*]. These findings provide culturally grounded evidence that extends prior conceptualizations of the construct (Bridges et al., 2012) and highlight its relevance for understanding prosocial and moral development in Latine youth (Calzada et al., 2010; Carlo & Conejo, 2019).

Importantly, these findings have particular relevance for adolescence, a developmental period characterized by increased autonomy, identity exploration, and engagement with social contexts beyond the family. During this time, culturally grounded values such as *bien educado* may serve as important guides for adolescents' social behavior, moral decision‐making, and interactions across contexts, including peer relationships and school environments. As youth navigate competing cultural expectations and develop a sense of self, parental socialization of *bien educado* may play a critical role in shaping how adolescents interpret and enact culturally valued norms of conduct.

The present findings both replicate and extend prior work on *bien educado* (Bridges et al., 2012). Several themes identified in the current study align with prior descriptions, particularly those related to respect, appropriate social conduct, and being well‐mannered. At the same time, the present study extends by highlighting how Latine mothers conceptualize *bien educado* as encompassing internal qualities, including *humildad* [humility] and *consideración hacia los demás* [consideration for others], which reflect empathy, moral reasoning, and relational awareness. In addition, *bien educado* was described as a broader evaluation of an individual's character and upbringing, emphasizing not only how individuals behave but how they are perceived by others as embodying culturally valued norms. Together, these findings suggest that *bien educado* operates at the intersection of observable behavior, internal moral qualities, and social evaluation, thereby expanding its placement within the broader nomological network of Latine cultural values and prosocial development.

As expected, *respeto* emerged as a key aspect of *bien educado*, serving as a marker of politeness and as a moral guide that influences children's behavior across contexts (Calzada et al., 2010; Halgunseth et al., 2006). This theme highlights *respeto* as an essential value socialized within the home and maintained generationally. Mothers viewed themselves as the primary agents responsible for fostering this value, reflecting culturally specific parenting approaches that support children's socialization within their families and the broader community. This emphasis on *respeto* highlights how Latine families socialize their children to interact positively with others, uphold family and cultural values, and navigate social interactions with dignity, courtesy, and sensitivity toward others.

An additional nuance that emerged from mothers' descriptions is that *bien educado* was often described as something that “comes from” the family, rather than something that is explicitly “taught.” This distinction suggests that the transmission of cultural values may be conceptualized as an embodied and relational process that is rooted in the family environment and modeled through everyday interactions. In this way, being *bien educado* may be viewed as a reflection of family identity and upbringing, where children internalize values through observation, immersion, and consistent exposure to parental practices.

A second key theme, *consideración hacia los demás* [consideration for others], highlights the importance of empathy and attentiveness to others' feelings, needs, and perspectives in social situations. Mothers often described this construct using more behaviorally grounded language, such as listening to others, following directions, and being attentive in social interactions. These descriptions align with commonly used expressions such as *hacer caso* or *prestar atención* (e.g., pay attention), suggesting that this theme reflects a broader interpretation of relational attentiveness rather than a direct use of the term *consideración* [consideration]. This emphasis reflects broader cultural expectations in Latine families, where warmth, responsibility, and maintaining positive relationships are associated with positive youth development outcomes (Carlo & Padilla‐Walker, 2020; Lopez et al., 2022). Mothers' perspectives align with research on collectivistic and relational cultural orientations, emphasizing interdependence, understanding, and responsiveness to others' needs (Campos et al., 2014). Their descriptions also suggest that teaching children to be considerate goes beyond politeness, but about preparing them to engage in respectful and supportive environments such as family, school, and community settings. By presenting consideration for others as a core aspect of being *bien educado*, mothers emphasize how this value functions as a cultural mechanism through which children learn to show warmth, respect, and relational norms characteristic of many Latine families (Campos et al., 2014).

The current findings also identified *humildad* (humility) as a core element of being *bien educado*. Mothers perceived humility as being receptive to others regardless of their background. Humility was deemed as demonstrating empathy and modesty when interacting with others, regardless of one's own status or accomplishments. This perspective reinforces how humility is deeply embedded in cultural expectations about social behavior and how mothers consider it as vital to their children's development, not only as individuals but as members of a larger, connected community. These findings are consistent with prior research on U.S. Mexican cultural values, specifically familism (e.g., family support, obligation, and referents), which emphasizes prioritizing family relationships and fulfilling responsibilities to others within the family (Knight et al., 2010; Schwartz et al., 2010).

A fourth theme emphasized by mothers' behaviors was being a well‐mannered person. For example, mothers described manners as more than just etiquette. They were described as active expressions of respect and empathy. Manners demonstrated children's ability to greet others appropriately, communicate respectfully, and behave warmly were viewed by mothers as direct indicators of a good moral upbringing. The current findings suggest, for example, that these behaviors serve as social capital, helping children succeed and gain acceptance in various settings, including those outside their cultural communities.

### Exploratory observations by language use

We also considered whether mothers' conceptions of *bien educado* varied by language use; however, these observations were exploratory given the small number of English‐dominant mothers. Interestingly, we could not discern any such notable differences. Instead, variation appeared in how values were described. For example, some mothers emphasized teaching *bien educado* through everyday actions like greeting adults, helping others, and being polite at home and in public. Others described these values in terms of empathy, fairness, and understanding other people's experiences in school and social situations. These patterns may suggest that language use may shape how cultural values are expressed, although further research is needed to better understand these preliminary observations and to better understand how bilingual and bicultural environments influence core value conceptualizations and socialization in Latine families.

More broadly, these findings contribute to existing research on Latine parenting by further highlighting how culturally grounded values shape parenting practices and youth development (Carlo & Conejo, 2019). Prior work has emphasized the role of values such as respect and familism in guiding children's social and moral development (Calzada et al., 2010; Knight et al., 2010). The present study extends this literature by highlighting *bien educado* as a culturally embedded construct that integrates these values and reflects how parents define and evaluate appropriate behavior and character. Importantly, assessing *bien educado* may provide a culturally responsive approach to understanding parenting practices and youth development in Latine families, particularly in contexts where traditional Western frameworks may not fully capture culturally specific socialization goals.

### Study limitations

There were several study limitations. First, the qualitative approach and sample size limit the generalizability of the findings. Studies of larger and more representative samples, as well as quantitative studies, could enhance our understanding of this important concept. Second, the grouping by language dominance was exploratory and requires additional research to draw stronger conclusions. Future research should include fathers and other caregivers and examine variations across different Latine subgroups (e.g., Cubans, Puerto Ricans). Third, longitudinal studies are needed to explore whether and how these cultural values change over time and in various settings. Fourth, demographic information such as income and marital status was not collected, which may limit the interpretation of socioeconomic influences on the findings. Finally, a potential limitation and future direction is that the present study focused solely on *bien educado* rather than examining *mal educado*. The interview questions were explicitly designed to understand the concept of *bien* educado. Therefore, references to *mal* educado emerged less frequently and were not systematically explored in depth, although the antithesis concept of *mal educado* was occasionally mentioned by participants. Future studies designed to examine *mal educado* and the relations between *bien educado* and *mal educado* are desirable to determine possible important distinctions. It is important to understand that *mal educado* might not simply be the opposite of *bien educado*, but rather a distinct construct. Furthermore, *mal educado* might not necessarily indicate a lack of proper moral socialization. For example, youth who appear anxious, withdrawn, or reserved may be misinterpreted as being impolite or disrespectful or *mal educado*, when in fact, these behaviors could be the result of shyness, anxiety, or situational influences (e.g., peer pressure).

## CONCLUSION

The primary aim of this study was to explore how Latine mothers conceptualized and defined the cultural value of *bien educado*, a multifaceted construct. The findings demonstrated that Latine mothers conceptualized *bien educado* as consisting of four main aspects: respect, consideration for others, humility, and being well‐mannered. Given the centrality of these four elements in prosocial and moral development (Carlo, 2014), the findings situate *bien educado* as a Latine‐grounded value that can further our understanding of U.S. Latine youth. Additionally, the findings emphasize the importance of including this understudied core Latine cultural value in sociocultural and ecologically grounded parental socialization models often overlooked in mainstream developmental and educational research, especially when studying U.S. Latine youth. The findings also challenge deficit‐oriented narratives about Latine parenting by highlighting the cultural strengths, intentionality, and pedagogical wisdom present in everyday family practices. These findings have significant implications for developing culturally grounded measures of *bien educado* and culturally responsive educational, clinical, and community‐based interventions that foster prosocial and moral development in Latine youth. Such efforts would deepen our understanding of how values like *bien educado* serve as both cultural assets and adaptive resources for Latine families navigating bicultural and bilingual contexts.

## AUTHOR CONTRIBUTIONS

All authors contributed substantially to the development of this manuscript and approved the final submitted version. Alysia Cruz conceptualized the study, designed the focus group protocol, led data collection, and conducted the primary analyses and interpretation. Gustavo Carlo provided conceptual guidance, contributed to the development of the research design, and offered feedback on the interpretation of findings. All authors contributed to writing and revising the manuscript and approved the final version for submission.

## FUNDING INFORMATION

The authors have nothing to report.

## CONFLICT OF INTEREST STATEMENT

The authors declare no conflict of interest.

## ETHICS APPROVAL

The research conducted in this study was reviewed and approved by the Institutional Review Boards at the University of California, Irvine. Approved October 2024. Reference Number: IRB: #5478. All aspects of the study complied with APA Ethical Principles. The manuscript has been approved by all authors and has not been published or reviewed for publication elsewhere.

## PATIENT CONSENT STATEMENT

All participants gave written informed consent as appropriate, and all participants were compensated for their time.

## Data Availability

The data are not publicly available due to privacy or ethical restrictions. Data may be available upon request from the corresponding author.

## APPENDIX 1 English Focus Group Interview Transcript

English focus group script and questions.

**Instructions:** *I am going to record our conversations so that I can listen to them afterwards. I hope you don't mind. I need to record it so that I can analyze the data for my thesis. Just know that I and my professor will be the only ones to listen to these recordings. Also, none of your personal information is identifiable and all of your responses are confidential. I really appreciate your honest opinions and answers to these questions. I really would like to hear from everyone—remember there are no right or wrong answers and I would really appreciate it if you would not interrupt each other while someone is speaking. Any questions before we start?*

**Introduction:** *Let's begin by discussing the value of bien educado. As many of you already know, bien educado refers to a person who is well‐mannered, demonstrating positive social behaviors, positive self‐conduct, and having a strong moral character. It is a broader construct that encompasses other Latine cultural values of respect, consideration for others, and being humilde (humble). Ok, you can tell by now that I am very interested in understanding the concept of bien educado and I would love to ask you all some questions to further understand the concept of bien educado. So, let me start by asking each of you:*

1. What is your own definition of *bien educado*?
  - Thank you
2. Now I would like to ask each of you how important is bien educado?
  - Thank you
3. How do you know that your child is bien educado?
4. What are some things that you do to teach your child to be bien educado?
  - Ok, now we have talked about the concept of being *bien educado*. What about the concept of being *mal educado*?
5. What does it mean to be mal educado?

**Conclusion:** *Ok, that is it. I want you to understand that I greatly appreciate your help and willingness to participate in my project. These conversations are going to enrich our understanding of this important concept that exists in our culture and will help us to understand how we can encourage our children to grow up to be bien educado. Thank you very much. My contact information is at the top of this form. Please feel free to contact me* via *phone or email*.

educado?
